# Detection of Suicidal Ideation in Clinical Interviews for Depression Using Natural Language Processing and Machine Learning: Cross-Sectional Study

**DOI:** 10.2196/50221

**Published:** 2023-12-01

**Authors:** Tim M H Li, Jie Chen, Framenia O C Law, Chun-Tung Li, Ngan Yin Chan, Joey W Y Chan, Steven W H Chau, Yaping Liu, Shirley Xin Li, Jihui Zhang, Kwong-Sak Leung, Yun-Kwok Wing

**Affiliations:** 1 Li Chiu Kong Family Sleep Assessment Unit, Department of Psychiatry, The Chinese University of Hong Kong, Hong Kong, China (Hong Kong); 2 Department of Psychology, The University of Hong Kong, Hong Kong, China (Hong Kong); 3 The State Key Laboratory of Brain and Cognitive Sciences, The University of Hong Kong, Hong Kong, China (Hong Kong); 4 Guangdong Mental Health Center, Guangdong General Hospital and Guangdong Academy of Medical Sciences, Guangdong, China; 5 Department of Computer Science and Engineering, The Chinese University of Hong Kong, Hong Kong, China (Hong Kong); 6 Department of Applied Data Science, Hong Kong Shue Yan University, Hong Kong, China (Hong Kong)

**Keywords:** depression, suicidal ideation, clinical interview, machine learning, natural language processing, automated detection

## Abstract

**Background:**

Assessing patients’ suicide risk is challenging, especially among those who deny suicidal ideation. Primary care providers have poor agreement in screening suicide risk. Patients’ speech may provide more objective, language-based clues about their underlying suicidal ideation. Text analysis to detect suicide risk in depression is lacking in the literature.

**Objective:**

This study aimed to determine whether suicidal ideation can be detected via language features in clinical interviews for depression using natural language processing (NLP) and machine learning (ML).

**Methods:**

This cross-sectional study recruited 305 participants between October 2020 and May 2022 (mean age 53.0, SD 11.77 years; female: n=176, 57%), of which 197 had lifetime depression and 108 were healthy. This study was part of ongoing research on characterizing depression with a case-control design. In this study, 236 participants were nonsuicidal, while 56 and 13 had low and high suicide risks, respectively. The structured interview guide for the Hamilton Depression Rating Scale (HAMD) was adopted to assess suicide risk and depression severity. Suicide risk was clinician rated based on a suicide-related question (H11). The interviews were transcribed and the words in participants’ verbal responses were translated into psychologically meaningful categories using Linguistic Inquiry and Word Count (LIWC).

**Results:**

Ordinal logistic regression revealed significant suicide-related language features in participants’ responses to the HAMD questions. Increased use of anger words when talking about work and activities posed the highest suicide risk (odds ratio [OR] 2.91, 95% CI 1.22-8.55; *P*=.02). Random forest models demonstrated that text analysis of the direct responses to H11 was effective in identifying individuals with high suicide risk (AUC 0.76-0.89; *P*<.001) and detecting suicide risk in general, including both low and high suicide risk (AUC 0.83-0.92; *P*<.001). More importantly, suicide risk can be detected with satisfactory performance even without patients’ disclosure of suicidal ideation. Based on the response to the question on hypochondriasis, ML models were trained to identify individuals with high suicide risk (AUC 0.76; *P*<.001).

**Conclusions:**

This study examined the perspective of using NLP and ML to analyze the texts from clinical interviews for suicidality detection, which has the potential to provide more accurate and specific markers for suicidal ideation detection. The findings may pave the way for developing high-performance assessment of suicide risk for automated detection, including online chatbot-based interviews for universal screening.

## Introduction

Up to 77% of individuals who died by suicide had contact with their primary care provider within 12 months prior to their death [1]. Suicidal ideation is a significant risk factor for suicidal death and is an important clinical concern [2-4]. Screening for suicidal ideation is a standard practice in health care settings. However, a past study reported poor agreement in rating suicide risk among primary care providers [5]. Assessing patients’ suicide risk is challenging, especially among those who deny suicidal ideation and perceive suicide as taboo to talk about [6-8]. In recent years, researchers have attempted to screen suicide risk unobtrusively based on implicit language-based clues in patients’ speech [9,10]. A study applied a naive Bayes classifier to patients’ verbal responses in clinical interviews to detect suicide risk with an area under the curve (AUC) of 0.63 [9]. This line of research is supported by recent behavioral and neuroimaging studies that demonstrated a close relationship between language use and social-emotional processing [11].

A systematic review has suggested that first-person singular pronouns and negative emotion words are language features of people who are suicidal [12]. However, first-person singular pronouns and negative emotion words are also found among those who are prone to depression [13,14], and first-person singular pronoun use has been proposed as a specific language marker of depression in a meta-analysis [15]. This result is concordant with the idea of depressive self-focus and rumination on past events, particularly on negative memories in depression. Given the overlapping language features among patients with depression and those with suicide risk, there is a need to look for language features that are specific in predicting suicide among patients with depression. Previous studies reported other linguistic features (eg, prepositions and verbs) for predicting suicide risk [12,16]. Another study differentiating suicide notes from depression notes and neutral blog posts found adverbs, cognitive processing words, and death words as the most significant language features of suicidality [17].

Natural language processing (NLP) and machine learning (ML) have been used to detect suicide risk [18,19]. Previous studies have incorporated NLP and ML into suicide detection for universal screening on social media [20-22]. Researchers have identified explicit suicide expressions (eg, suicide notes) [23] and texts written by suicidal individuals (eg, songs, poems, diaries) [24], but these have mostly been searched for in social media posts and other personal documents [25]. Few studies have evaluated the techniques in clinical settings [18,19,26]. This study used a text analysis approach using NLP and ML techniques for suicidal ideation detection from the words spoken by participants in clinical interviews for depression. It is hypothesized that (1) language features extracted from responses to interview questions are associated with suicidal ideation; (2) there is a difference in language features between suicidal ideation and depression; and (3) the model can contribute to more accurate suicidal ideation detection, especially among patients with depression.

## Methods

### Recruitment

This cross-sectional study was part of an ongoing digital phenotyping research project in characterizing depression with a case-control design [27]. Patients with lifetime major depressive disorder (MDD) were recruited from outpatient clinics in a local university-affiliated hospital. The diagnosis of any psychiatric disorder was made by the attending psychiatrist. Controls were recruited from the community. The Mini-International Neuropsychiatric Interview, version 5.0, was used to check if they had any *Diagnostic and Statistical Manual of Mental Disorders*, fourth edition, diagnosis [28]. We found that 8% (9/117) of the community sample had lifetime MDD, and these individuals were considered as cases. The diagnostic criteria for MDD included having (1) 5 or more depressive symptoms for ≥2 weeks, (2) either depressed mood or loss of interest and pleasure, (3) symptoms causing significant distress or impairment, and (4) no manic or hypomanic behavior. Participants who, at some point in their lives, had ever received a diagnosis were classified as cases of lifetime MDD. Inclusion criteria for participation were (1) being a native Cantonese speaker and (2) being a Chinese adult aged 18 to 65 years. Participants (1) with any voice, speech, and language problems; (2) with any history of psychiatric disorder other than MDD; and (3) who were incompetent to give written informed consent were excluded. The data collection was conducted between October 2020 and May 2022. This study included 197 cases and 108 controls (n=305).

### Ethical Considerations

Ethical approval was obtained from the Joint Chinese University of Hong Kong–New Territories East Cluster clinical research ethics committee (2020.492).

### Clinical Measurements

The structured interview guide for the Hamilton Depression Rating Scale (HAMD) was adopted [29]. The HAMD-17 shows high interrater reliability [30]. The interviews were conducted by the second author (JC), who holds an MD and a PhD. Each interview took 15 to 30 minutes. We sequentially probed 14 questions on the HAMD-17 (H1 to H14) to assess depression symptoms (we rated questions H15 to H17 based on observation during the interview). Depression severity was classified as no depression (score 0-7), mild depression (8-16), and moderate to severe depression (≥17) [29].

H11, which was used to assess suicide risk, asks “Since last week, have you had any thoughts that life is not worth living?” Suicide risk was rated in five progressive levels: (1) having no suicidal thoughts; (2) feeling life is not worth living; (3) having wishes to be dead, or any thoughts of possible death of self; (4) having suicidal ideation or gestures; and (5) having attempts at suicide. JC and TMHL rated H11 with high interrater reliability (κ=0.92). Discrepancies in ratings were discussed until a consensus was reached. A total of 236 of 305 participants (77%) who did not report any suicidal thoughts were classified as the nonsuicidal group. The severity of suicidal ideation ranged from passive ideation (ie, having wishes to be dead) to active ideation (ie, having suicidal thoughts and behaviors); persons with active ideation are more prone to suicide attempts and deaths than those with passive ideation [3]. Thus, 33 and 23 participants who felt life was not worth living and had wished to be dead, respectively, were classified as the low-suicide-risk group (18%); 12 participants and 1 participant who had suicidal thoughts and acting out behavior, respectively, were classified as the high-suicide-risk group (4%). Multimedia Appendix 1 includes verbatim quotations of the verbal responses to H11.

### Feature Extraction

The interviews were recorded and transcribed by a research assistant with a psychology background. The transcripts were checked by the first author (TMHL). As interword spacing is absent in Chinese texts (eg, “I want to kill myself” in Chinese would become “Iwanttokillmyself”), Chinese word segmentation was needed to separate words. For Chinese word segmentation, the study used a deep learning–based Chinese word segmentation engine, fastHan, which included local text samples for training and testing its segmentation model, achieving over 90% agreement with human segmentation [31].

Words were translated into psychologically meaningful categories using Linguistic Inquiry and Word Count (LIWC) [32]. There are 71 categories in the Chinese version of LIWC [33]. To investigate if a language feature *f* existed within a verbal response, we calculated the proportion of words *r_i_* in the response that matched with any of the words *c_j_* listed in an LIWC category using the following formula:


$$f=\frac{\sum_{\;r_{i}\;\in \;R,\;\;\;c_{j}\;\in \;C}m\;\left(r_{i}\;,\;c_{j}\right)}{|R|}$$

where *R*={*r_1_*, *r_2_*, …} and *C*={*c_1_*, *c_2_*, …} denotes the collection of words in the response and the LIWC category, respectively, while *m(r, c*) represents checking for an exact match between *r* and *c* (which returns 1=matched or 0=not matched), and |*R*| denotes the number of words in the response. All the features were calculated by the proportion of words of each category relative to text length (as a percentage). Using the relative frequency minimized the confounding factor of text length in interview responses.

### Statistical Analysis

All analyses were conducted using R (version 4.2.0; R Foundation for Statistical Computing). A *P* value <.05 was considered statistically significant. Descriptive statistics for continuous variables are shown as means and SDs, while categorical variables are presented as numbers and percentages. Age and the number of words in interview responses were compared among the nonsuicidal, low-suicide-risk, and high-suicide-risk groups using a 1-way ANOVA, while gender and depression severity were compared with the chi-square test among the groups. The Cramér *V* was used to measure the associations of clinician ratings of the HAMD questions with suicide risk.

Ordinal logistic regression was performed to model the relationship between language features (predictors) and suicide risk (the outcome)—ordered in three progressive levels from (1) nonsuicidal, (2) low suicide risk, to (3) high suicide risk [34]. The analysis was conducted for the responses to H11 (a suicide-related question) and the responses to other questions (content without suicide disclosure). The regression models were adjusted for age, gender, and depression severity. Adjusted odds ratios (ORs) with 95% CIs were calculated as a measure of the strength of association. The ORs were standardized across the HAMD questions and visualized using a heatmap. Euclidean distance was used as the similarity measure for clustering the questions with similar language features associated with suicide risk. Bonferroni correction for multiple testing was applied.

Random forest, an ML classification technique [35], was used to detect participants with suicide risk, including (1) high suicide risk and (2) any suicide risk (both low and high risk) among (1) all participants, (2) participants with lifetime MDD, (3) participants with lifetime MDD and unremitted depression (HAMD-17 score ≥8), (4) participants with lifetime MDD and remitted depression, and (5) control participants, based on language features extracted from their interview responses. All classification results were evaluated by leave-one-out cross-validation. Receiver operating characteristic curve analysis was used for analyzing the accuracy of classification results. Statistics included the AUC and 95% CI, sensitivity, and specificity of the ML classifiers. For each classifier, sensitivity and specificity at the optimal cutoff were computed.

## Results

### Characteristics of the Participants

Table 1 shows the characteristics of the 3 groups, namely the nonsuicidal, low-suicide-risk, and high-suicide-risk groups. No significant age or gender differences were found among the 3 groups. Suicide risk was correlated with depression severity (*V*=0.45, 95% CI 0.36-0.52; *P*<.001). All participants in the high-suicide-risk group were depressed, of which the majority (8/13, 62%) were moderately to severely depressed. In the low-suicide-risk group, 86% (48/56) had depression. The majority (28/56, 50%) were experiencing mild depression. Only 23% (54/236) of nonsuicidal participants had depression, most of whom (46/236, 20%) had mild depression. Participants, on average, generated 387.93 (SD 349.19) words in response to all 14 interview questions. There were significant differences among the 3 groups in terms of the number of words in their interview responses. The low-suicide-risk group generated longer responses than the nonsuicidal group (*P*<.001) and high-suicide-risk group (*P*=.02). The high-suicide-risk group also uttered marginally more words compared to the nonsuicidal group (*P*=.05).

**Table 1.** Characteristics of the participants (N=305).

Characteristics		Nonsuicidal (n=236)	Low suicide risk (n=56)	High suicide risk (n=13)	*P* value
Age (years), mean (SD)		52.63 (11.57)	54.46 (11.88)	51.69 (15.42)	.27
**Gender, n (%)**					.37
	Female	132 (56)	37 (66)	7 (54)	
	Male	104 (44)	19 (34)	6 (46)	
**Lifetime major depressive disorder, n (%)**					<.001
	Yes	133 (56)	51 (91)	13 (100)	
	No	103 (44)	5 (8)	0 (0)	
**Depression severity^a^, n (%)**					<.001
	None	182 (77)	8 (14)	0 (0)	
	Mild	46 (20)	28 (50)	5 (38)	
	Moderate or severe	8 (3)	20 (36)	8 (62)	
**Number of words in responses, mean (SD)**					
	To all questions except H11^b^	313.86 (280.60)	604.57 (434.45)	420.08 (187.05)	<.001
	To H11 only	7.91 (16.78)	48.77 (58.96)	25.62 (18.89)	<.001

a Severity ranges for the Hamilton Depression Rating Scale–17: no depression (0-7); mild depression (8-16); moderate to severe depression (≥17).

b H11: Hamilton Depression Rating Scale question 11.

Table 2 shows the associations of clinician ratings of the HAMD questions with suicide risk. All the ratings were associated with suicide risk (*V*=0.14-0.40). The clinician rating of H12 (on anxiety psychic) had the strongest association with suicide risk, whereas the rating of H8 (on insomnia late) had the weakest association with suicide risk.

**Table 2.** Associations of clinician ratings of the Hamilton Depression Rating Scale (HAMD) questions with suicide risk (based on H11).

HAMD item	Question type	Cramér *V* (95% CI)	*P* value
H1	Depressed mood	0.36 (0.26-0.42)	<.001
H2	Work and activities	0.36 (0.27-0.43)	<.001
H3	Genital symptoms	0.21 (0.12-0.28)	<.001
H4	Somatic symptoms gastrointestinal	0.30 (0.21-0.37)	<.001
H5	Loss of weight	0.18 (0.08-0.25)	.005
H6	Insomnia early	0.23 (0.13-0.30)	<.001
H7	Insomnia middle	0.17 (0.07-0.24)	.003
H8	Insomnia late	0.14 (0.01-0.20)	.03
H9	Somatic symptoms general	0.30 (0.21-0.37)	<.001
H10	Feelings of guilt	0.36 (0.26-0.43)	<.001
H12	Anxiety psychic	0.40 (0.30-0.46)	<.001
H13	Anxiety somatic	0.31 (0.22-0.38)	<.001
H14	Hypochondriasis	0.28 (0.18-0.34)	<.001

### Associations of Suicide Risk With Language Features in H11

Suicide risk was assessed by clinicians based on H11 (a suicide-related question). Table 3 shows the significant associations of suicide risk with language features in verbal responses to H11. Suicide risk was positively associated with linguistic (ie, function words, verbs, adverbs, prepositions, and numbers), psychological (ie, social, cognitive, and biological process words, relativity words, death words, and fillers), and Chinese-specific categories (ie, postpositions, quantity units, multifunction words, and tense markers). After adjusting the analyses for depression severity, 11 language features remained significant. Among the 11 features, increased use of past tense markers posed the highest suicide risk. For every 1% increase in past tense markers, the odds of being more likely to have higher suicide risk (low or high suicide risk vs nonsuicidal) were multiplied 1.24 times (OR 1.24, 95% CI 1.09-1.43; *P*=.002).

**Table 3.** Significant associations of suicide risk with language features in verbal responses to H11 (Hamilton Depression Rating Scale question 11) using ordinal logistic regression.

LIWC^a^ category	Nonsuicidal (n=236), mean (SD)	Low suicide risk (n=56), mean (SD)	High suicide risk (n=13), mean (SD)	Odds ratio (95% CI)^b^	*P* value	Odds ratio (95% CI)^c^	*P* value
Function words	19.48 (22.32)	42.48 (15.75)	44.25 (17.64)	1.05 (1.03-1.07)	<.001	1.04 (1.02-1.06)	<.001
Verbs	3.30 (10.62)	15.08 (10.11)	21.90 (8.03)	1.13 (1.09-1.16)	<.001	1.08 (1.05-1.11)	<.001
Auxiliary verbs	0.39 (1.68)	4.14 (7.46)	5.27 (4.12)	1.28 (1.18-1.40)	<.001	1.17 (1.08-1.28)	<.001
Adverbs	7.10 (14.73)	11.59 (9.06)	11.06 (8.14)	1.02 (1.00-1.04)	.03	1.02 (1.00-1.05)	.07
Prepositions	0.92 (2.85)	7.07 (7.52)	12.22 (10.04)	1.24 (1.18-1.31)	<.001	1.19 (1.13-1.26)	<.001
Numbers	0.32 (2.48)	1.70 (4.72)	0.48 (1.37)	1.09 (1.01-1.18)	.02	1.03 (0.94-1.11)	.45
Postpositions	0.86 (3.64)	3.15 (5.31)	2.22 (3.58)	1.10 (1.04-1.16)	.002	1.06 (1.00-1.13)	.06
Quantity units	1.06 (4.04)	3.05 (3.56)	1.53 (2.54)	1.09 (1.02-1.16)	.009	1.04 (0.96-1.12)	.32
Multifunction words	1.98 (9.79)	8.75 (9.70)	11.11 (8.70)	1.09 (1.05-1.13)	<.001	1.05 (1.03-1.09)	<.001
Tense markers	0.30 (1.87)	1.52 (2.88)	3.09 (4.27)	1.30 (1.16-1.46)	<.001	1.15 (1.03-1.29)	.01
Past tense markers	0.15 (1.25)	1.09 (2.45)	2.43 (3.99)	1.40 (1.22-1.63)	<.001	1.24 (1.09-1.43)	.002
Present tense markers	0.00 (0.00)	0.23 (0.94)	0.00 (0.00)	1.71 (1.08-2.90)	.02	1.07 (0.65-1.78)	.80
Social processes	0.60 (2.35)	1.74 (3.12)	0.39 (0.95)	1.10 (1.00-1.20)	.04	0.93 (0.82-1.04)	.22
Family	0.02 (0.26)	0.37 (1.10)	0.00 (0.00)	1.66 (1.15-2.41)	.006	1.04 (0.70-1.54)	.86
Cognitive processes	13.89 (19.00)	20.12 (14.96)	18.05 (12.42)	1.02 (1.00-1.03)	.02	1.01 (0.99-1.03)	.16
Discrepancy	0.94 (4.62)	4.33 (7.42)	5.74 (4.38)	1.12 (1.06-1.19)	<.001	1.08 (1.03-1.14)	<.001
Tentative	1.16 (3.96)	5.41 (7.76)	2.96 (2.82)	1.10 (1.06-1.16)	<.001	1.05 (1.00-1.11)	.04
Biological processes	0.14 (0.92)	1.01 (2.36)	1.04 (3.10)	1.34 (1.16-1.58)	<.001	1.04 (0.87-1.23)	.69
Body	0.09 (0.64)	0.45 (1.15)	0.83 (2.35)	1.55 (1.21-2.02)	<.001	1.04 (0.77-1.41)	.79
Health	0.05 (0.37)	0.56 (2.03)	0.21 (0.77)	1.32 (1.08-1.74)	.01	1.03 (0.83-1.32)	.76
Relativity	2.23 (6.13)	9.21 (9.77)	7.23 (7.26)	1.09 (1.06-1.13)	<.001	1.05 (1.02-1.09)	.004
Motion	0.73 (3.47)	2.14 (3.18)	1.09 (1.79)	1.08 (1.01-1.16)	.03	1.03 (0.93-1.11)	.55
Space	0.88 (3.64)	2.38 (3.41)	3.85 (4.41)	1.12 (1.04-1.21)	.002	1.07 (0.99-1.15)	.07
Time	1.12 (3.87)	6.22 (9.16)	4.03 (5.72)	1.10 (1.06-1.15)	<.001	1.06 (1.01-1.11)	.01
Death	0.11 (1.32)	0.68 (1.89)	0.83 (2.35)	1.27 (1.06-1.55)	.02	1.12 (0.93-1.30)	.18
Filler	0.71 (2.64)	1.57 (2.80)	1.57 (3.31)	1.10 (1.01-1.20)	.03	1.01 (0.90-1.12)	.88

a LIWC: Linguistic Inquiry and Word Count.

b Adjusted for age and gender.

c Adjusted for age, gender, and depression severity.

### Associations of Suicide Risk With Language Features Across Other HAMD Questions

**Figure 1.**
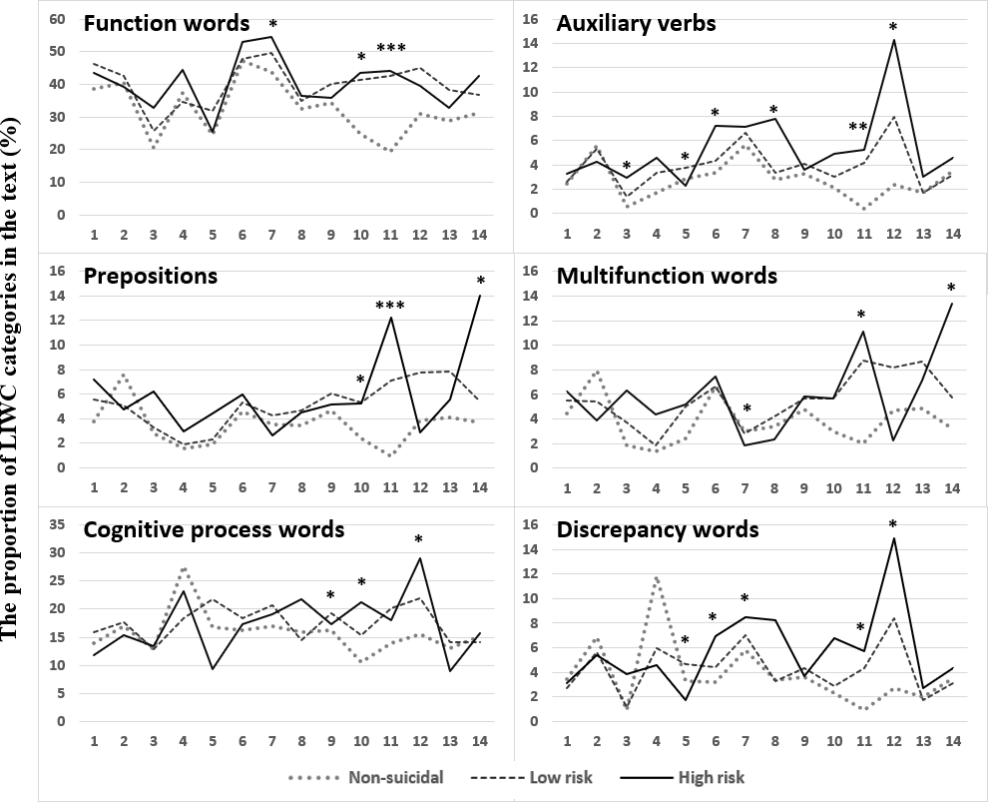
Significant suicide-related features of language vary across the Hamilton Depression Rating Scale questions H1-14. Six language features (namely function words, auxiliary verbs, prepositions, multifunction words, cognitive process words, and discrepancy words) were found to be associated with suicide risk in 3 or more questions. LIWC: Linguistic Inquiry and Word Count. *P<.05; **P<.01; ***P<.001.

Suicide-related language features varied across other HAMD questions (see Multimedia Appendix 2). Among the language features, increased use of anger words in H2 posed the highest suicide risk. For every 1% increase in anger words, the odds of being more likely to have suicide risk were multiplied 2.91 times (OR 2.91, 95% CI 1.22-8.55; *P*=.02). Some language features, on the other hand, were negatively associated with suicide risk. For example, increased use of feeling words (OR 0.36, 95% CI 0.12-0.82; *P*=.04), future tense markers (OR 0.50, 95% CI 0.23-0.84; *P*=.03), and anxiety words (OR 0.57, 95% CI 0.29-0.88; *P*=.04) when responding to H2, H8, and H13, respectively, reduced suicide risk. Overall, six language features—namely function words, auxiliary verbs, prepositions, multifunction words, cognitive process words, and discrepancy words—were found to be associated with suicide risk in 3 or more questions; these are illustrated in Figure 1.

**Figure 2.**
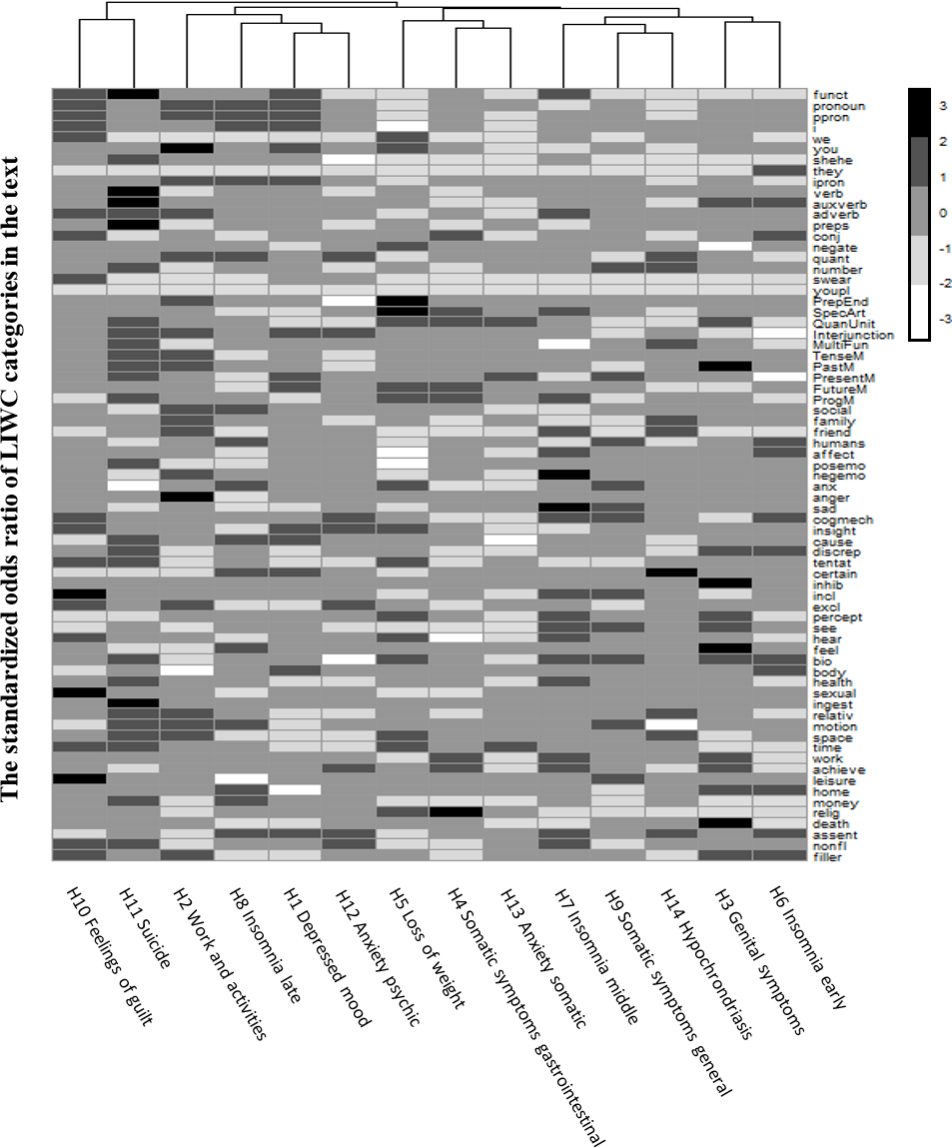
Heatmap of associations between language features and suicide risk across the Hamilton Depression Rating Scale (HAMD) questions (H1-14, where H11 asks about suicidal ideation) in all participants (n=305). The heatmap shows the standardized odds ratios (darker colors represent greater associations with suicide risk) obtained from ordinal logistic regression models adjusted for age, gender, and depression severity. Euclidean distance was used as the similarity measure for clustering the HAMD questions with similar language features associated with suicide risk shown in the dendrogram. LIWC: Linguistic Inquiry and Word Count.

Figure 2 illustrates the hierarchical relationship between the HAMD questions based on the association of language features with suicide risk. Questions with similar suicide-related language features are closer and joined as clusters in the dendrogram. For instance, H10 (asking about feelings of guilt) joined together with H11 (a suicide-related question) as a cluster. While the heights reflect the similarity between the clusters, the largest difference between clusters is between the clusters of H10 and H11 vs the clusters of the other questions. Some possible clusters were observed in the dendrogram, including (1) the suicide-related cluster: H10 and H11; (2) the mood cluster: H2, H8, H1, and H12; (3) the appetite cluster: H5, H4, and H13; (4) the somatic symptom cluster: H7, H9, and H14; and (5) the sexual activity cluster: H3 and H6.

### The Performance of Suicide Risk Detection

**Figure 3.**
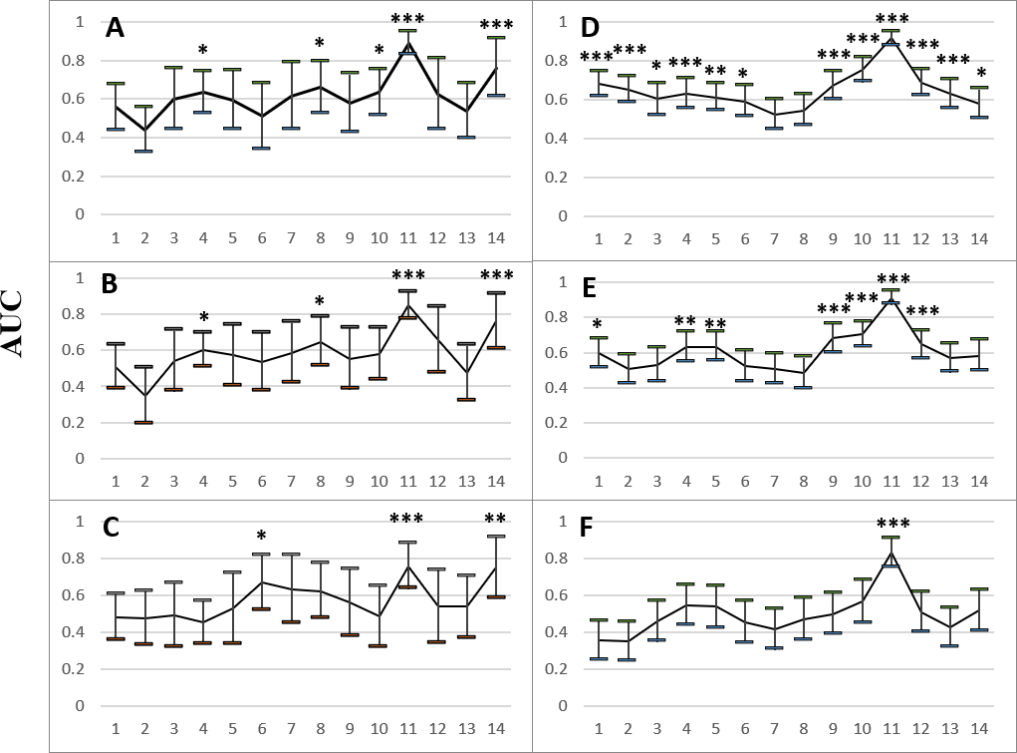
The performance, in terms of area under the curve (AUC), for detecting (A-C) active and (D-F) active and passive suicidal ideation based on participants’ verbal responses to the Hamilton Depression Rating Scale (HAMD) questions H1 to H14 (where H11 asks about suicidal ideation) using random forest with leave-one-out cross-validation. Participants with active ideation (n=13) were detected among (A) all participants (n=305), (B) those with lifetime major depressive disorder (MDD; n=197), and (C) those with lifetime MDD and HAMD-17 score ≥8 (n=109). Participants with active or passive ideation were detected (n=69, 64, and 60, respectively) among (D) all participants, (E) those with lifetime MDD, and (F) those with lifetime MDD and HAMD-17 score ≥8. The error bars represent the 95% CI for AUC. *P<.05; **P<.01; ***P<.001.

Figure 3 depicts the performance for detecting suicide risk based on verbal responses to the HAMD questions in the ML analysis. Based on the responses to H11 (the outcome measure), ML models were trained to identify individuals with high risk in (Figure 3A) all participants, (Figure 3B) those with lifetime MDD, and (Figure 3C) those with lifetime MDD and unremitted depression (AUC 0.76-0.89; *P*<.001; sensitivity=0.69-0.85; specificity=0.73-0.84; Multimedia Appendix 3). H11 could also be used to detect suicide risk in general (including both high and low suicide risk) in (Figure 3D) all participants, (Figure 3E) those with lifetime MDD, and (Figure 3F) those with lifetime MDD and unremitted depression (AUC 0.83-0.92; *P*<.001; sensitivity=0.77-0.84; specificity=0.78-0.84). None of the control participants (without lifetime MDD) had high suicide risk, while 3 healthy controls reported low suicide risk. Using only H11, suicide risk could be detected among control participants (AUC 0.70; *P*=.04; sensitivity=0.80; specificity=0.71).

Based on the responses to H14 (on hypochondriasis), ML models were trained to identify individuals with high suicide risk in (Figure 3A) all participants (AUC 0.76; *P*<.001; sensitivity=0.69; specificity=0.70), (Figure 3B) those with lifetime MDD (AUC 0.76; *P*<.001; sensitivity=0.77; specificity=0.77), and (Figure 3C) those with lifetime MDD and unremitted depression (AUC 0.75; *P*=.003; sensitivity=0.69; specificity=0.69). H4, H6, H8, and H10 could also be used to detect high suicide risk. Except for H7 and H8, other questions could be used to detect suicide risk in general (including both high and low suicide risk) in (Figure 3D) all participants. Based on the responses to H1, H4, H5, H9, H10, and H12, ML models identified individuals with suicide risk in (Figure 3E) those with lifetime MDD.

## Discussion

### Overview

This study aimed to provide a novel perspective on suicidal ideation detection by analyzing texts in clinician-administered, structured interviews with NLP and ML. LIWC extracts human-understandable language features without knowing the interview content and can preserve patient privacy in clinical research. The study complements previous research with social media data (over 80% of the related studies were conducted using social media texts [19]) by (1) incorporating clinician-rated measures as the outcome, (2) investigating texts generated for specific purposes (ie, responding to the HAMD questions), and (3) exploring the added value of text analysis to clinical practice. The findings show that significant language features extracted from verbal responses to interview questions are associated with clinician-rated suicide risk. There is a difference in language features between suicidal ideation and depression. The ML models demonstrate that using direct responses to H11 is effective in identifying participants with suicide risk. More importantly, suicide risk can also be detected with satisfactory performance even without patients’ disclosure of suicidal ideation.

### Principal Results

The study distinguished suicidal ideation from its major confounding effect, depression, with an aim to investigate solely suicide-related language features. The use of past tense markers, verbs, and prepositions in response to a suicide-related question posed a higher risk than other language features. This finding on past tense markers is coherent with previous studies in which rumination occurred in suicidal individuals or past suicide attempts were described [12,15]. An increase in verbs is also found in previous research, which implies an aggravated suicide risk when suicidal intentions and thoughts become actions [14,16]. Prepositions are a relatively new language feature finding. This is consistent with a systematic review that analyzed 75 studies, only one of which reported increased use of prepositions of suicidal thoughts and behaviors [12]. In the modern Chinese language, neuroscience evidence suggests that prepositions are probably not a separate word class from verbs (the action words in a sentence) [36], which are also more frequently used by suicidal people. Another explanation for prepositions as connectors of words is that they demand and convey information about location, time, or direction [37]. An increase in prepositions use by suicidal people when responding to a suicide-related question may highlight the existence of concrete suicide plans.

Many previous depression and suicide detection studies were conducted on social media rather than clinical interviews [18,19]. Social media texts freely generated by users are often nonspecific to suicide or depression [22]. This study analyzed participants’ responses to the HAMD questions, specifically focusing on a series of depressive symptomatologies. Results were mixed for suicide-related language features in responses to different questions. Previous studies reported a significant difference in the use of first-person singular pronouns and negative emotion words between suicidal and nonsuicidal groups [12,24]. This study consistently found these patterns posed a higher suicide risk: increased use of anger words on the work and activities question (H2), negative emotion words on the middle insomnia question (H7), and sadness words on the somatic symptoms question (H9). However, the increased use of first-person singular pronouns and anxiety words when responding to H5 (on the loss of weight) and H13 (on anxiety somatic), respectively, were intriguingly found to reduce suicide risk, which seems contradictory to previous studies that found these 2 categories were major language features of suicidal ideation [12]. It is speculated that rather than being generic, suicide-related language features appear to be more topic specific. The use of first-person singular pronouns to describe feelings and daily activities could reflect self-focus, a low level of social integration, and even suicidal rumination [12]. Paying more attention to oneself in the context of body weight management (in H5), on the other hand, may indicate positive self-concept and self-compassion [38]. Using more anxiety words (in H13) to describe the relationship between somatic symptoms and anxiety may signify a good understanding of one’s own condition.

ML on the response to H11 (the outcome measure) is, understandably, highly effective in identifying both clinical and control participants with suicide risk, which is consistent with positive findings from previous studies detecting suicide-related social media posts labeled using human annotation [21,23]. With AUCs up to 92%, automatic detection of suicidal ideation in clinical settings, especially among busy and primary care clinics, seems possible. Real-time feedback from the ML models during clinical interviews can potentially facilitate early detection of suicidal ideation [8]. The ML models can be developed with other automated techniques, including chatbot-based interviews for future screening [10]. Furthermore, suicide risk can be detected with satisfactory performance without participants’ disclosure of suicidal ideation or attempts (eg, in H10 on feelings of guilt and H14 on hypochondriasis), which provides new evidence on the association of language use with suicidality. In particular, while the clinician rating of H14 is not the strongest predictor for suicide risk, the responses to H14, surprisingly, generate the most powerful language clues to identify high suicide risk in the ML analysis. Verbal responses tend to provide subtle yet more objective language-based information about underlying suicidal ideation, which transcends depressive symptomatologies. The findings suggest that the investigation of suicide-related language features should be an important research topic apart from studying the clinical correlates of suicidality. The evidence strengthens the use of NLP and ML for suicidal ideation detection using implicit language-based clues, especially when health care personnel cannot identify patients’ suicide risk upon a direct suicide-related question, or when patients refuse to answer a suicide-related question.

### Limitations

First, although we had a relatively large sample size of patients, there were few high-suicide-risk participants. The sample size of at-risk groups will be increased for a more balanced sample. Second, this study focused on a cross-sectional time frame to investigate the discriminating power of language features for suicide detection. A longitudinal study could contribute to a longer time-frame exploration of the temporal association of language use with suicidal thoughts and behaviors. Third, although the HAMD-17 showed high interrater reliability [30] and high intermethod reliability [39], and JC and TMHL rated H11 with high interrater reliability (κ=0.92), the reliability of the interview ratings of the rest of the HAMD questions in the current study was not assessed. H11 was used to determine whether the participants had suicide risk, while the text analysis was also conducted on the patient’s verbal responses in the interview. It is also difficult to avoid errors arising from participants’ willful denial of suicidal ideation or participants’ belief that talking about suicide is taboo. Future research may need a more comprehensive measure for the outcome of suicidality that is based on more objective benchmarks (eg, actual suicidal behaviors or history). Fourth, the current study did not compare various machine learning algorithms (eg, support vector machines, naive Bayes classifiers, and neural networks), model parameters, and feature sets to achieve the best results. The performance of suicidal ideation detection might be an underestimate. The paper also proposes future directions to use deep learning–based models, such as Bidirectional Encoder Representations from Transformers (BERT), MentalBERT, and other large language models [40] to conduct comparative experiments. Finally, the language and cultural differences between the Chinese and English languages, which could create deviations among the studies, should be investigated. Chinese people seem more conservative in expressing emotions, and topics like suicide may receive media airtime but not be present in their personal lives [6,7]. Only rarely do Chinese or, more broadly, Asian people express personal feelings and suicidal ideation, as they commonly see these as signs of a weak personality (eg, irresponsible, fragile, impulsive, and attention-seeking), which culminates and intertwines with the social stigma of mental disorders in the general population [41].

### Conclusions

The study investigated a novel perspective of using NLP and ML to analyze the texts from clinical interviews for suicidal ideation detection, which has the potential to provide more accurate and specific markers for suicide detection. Suicide risk detection is crucial groundwork for precrisis management to respond to safety concerns in automated screening and assessment. Other media such as chatbots and text-based detection have been used in many mental health applications. We hope that the enhanced suicide detection described in this study will augment and strengthen the performance of screening and assessment in the future.

## Supplementary material

10.2196/50221 Multimedia Appendix 1 Verbatim transcription of the responses to H11 among the 3 groups.

10.2196/50221 Multimedia Appendix 2 Significant associations of suicide risk with language features in verbal responses to the Hamilton Depression Rating Scale (HAMD) questions using ordinal logistic regression.

10.2196/50221 Multimedia Appendix 3 The performance of detecting suicide risk based on verbal responses to the Hamilton Depression Rating Scale (HAMD) questions using random forest with leave-one-out cross-validation.

## Abbreviations

AUC: area under the curve
BERT: Bidirectional Encoder Representations from Transformers
HAMD: Hamilton Depression Rating Scale
LIWC: Linguistic Inquiry and Word Count
MDD: major depressive disorder
ML: machine learning
NLP: natural language processing
OR: odds ratio
