# Supplementary material for: Detection of Suicidal Ideation in Clinical Interviews for Depression Using Natural Language Processing and Machine Learning: Cross-Sectional Study
Source: JMIR Med Inform. 2023 Dec 1;11:e50221. doi: 10.2196/50221 (PMC10718481; doi:10.2196/50221)
Supplement: Multimedia Appendix 1 [file medinform-v11-e50221-s001.docx]

**Table A1.** Verbatim transcription of the responses to H11 among the three groups.

| Verbatim quotations in the non-suicidal group | |
| --- | --- |
| 1. 唔會，生活好精彩。  哈哈哈，真係好精彩，人生好快樂㗎嘛。哈哈。 | 1. Nah, my life is such a blast!  Hahaha, truly amazing, life is full of blessings, innit? Haha. |
| 2. 生活冇乜意思啊，平淡算唔算？  唔，又唔係冇意思，淨係平淡。 | 2. Not really much of a purpose, bit boring and dull, do you know what I mean?  Hmm, not to the extent of not worthy, just unexciting. |
| 3. 諗吓先，呢個有冇……應該冇，印象、印象好似冇、冇呢樣嘢。印象冇呢樣嘢。 | 3. Well, 'bout this......not really, I don't reckon...don't think so, don't think I've thought 'bout it. |
| Verbatim quotations in the low-suicide-risk group | |
| 1. 咁生活係冇乜意思咁樣囉 ， 都真係有諗過早啲死囉。  但係冇話真係走去死。  咁我真係早啲死好過，成日都好似冇乜意思咁樣。 | 1. Life is a bit meaningless…have thought about it indeed.  But not that (I) would really do it…  It’s just…I don’t find any meanings, it’s better to just die earlier. |
| 2. 有啊有一次囉。又唔係真係好強烈嘅，即係有時都會諗吓做唔做人都冇乜所謂。 | 2. Yes, there was once. (Suicidal thoughts) Is not really so strong but like once in a while, (I) don’t find it matters to be alive or not. |
| Verbatim quotations in the high-suicide-risk group | |
| 1. 其實呃有試過諗過想死囉 。  上個星期我諗閃過一刻 。  即係有過嗰一刻好低落嘅時候，不如死咗佢 就算啦。 | 1. Indeed uh…have thought about dying.  Last week, (it) has flashed into my mind.  Like in that one moment of depressed (feelings), (I)’d rather just die. |
| 2.呃偶然會。  有𠝹手發洩。  上個禮拜。呃做咗之後跟住屋企人攔阻落嚟。 | 2. Uh...occasionally will.  Cut my wrist to vent.  Last week…uh…family members stopped (me) after what (I) did. |
