# Supplementary material for: Detection of Suicidal Ideation in Clinical Interviews for Depression Using Natural Language Processing and Machine Learning: Cross-Sectional Study
Source: JMIR Med Inform. 2023 Dec 1;11:e50221. doi: 10.2196/50221 (PMC10718481; doi:10.2196/50221)
Supplement: Multimedia Appendix 2 [file medinform-v11-e50221-s002.docx]

**Table A2.** Significant associations of suicide risk with language features in verbal responses to the HAMD questions using ordinal logistic regression.

| HAMD item | Question type | LIWC category | Non-suicidal  (n=236) | Low suicide risk  (n=56) | High suicide risk  (n=13) | OR (95% CI) | p |
| --- | --- | --- | --- | --- | --- | --- | --- |
| H2 | Work and activities | Past tense markers | 0.27 (0.95) | 0.63 (1.42) | 0.79 (1.89) | 1.30 (1.01-1.65) | .03 |
|  |  | Social processes | 1.39 (2.29) | 2.41 (2.67) | 2.54 (2.74) | 1.13 (1.00-1.28) | .04 |
|  |  | Anger | 0.02 (0.22) | 0.04 (0.25) | 0.35 (1.11) | 2.91 (1.22-8.55) | .02 |
|  |  | Feeling | 0.13 (0.58) | 0.11 (0.33) | 0.00 (0.00) | 0.36 (0.12-0.82) | .04 |
| H3 | Genital symptoms | Auxiliary verbs | 0.55 (2.32) | 1.35 (3.27) | 2.95 (5.93) | 1.12 (1.02-1.23) | .01 |
|  |  | Work | 0.07 (0.74) | 0.06 (0.31) | 1.10 (3.96) | 1.26 (0.98-1.63) | .04 |
| H4 | Somatic symptoms gastrointestinal | Specific attributives | 0.83 (3.28) | 0.85 (3.08) | 1.92 (4.32) | 1.10 (0.99-1.20) | .04 |
| H5 | Loss of weight | First-person singular pronouns | 2.70 (4.91) | 2.33 (3.92) | 2.10 (3.83) | 0.89 (0.81-0.97) | .01 |
|  |  | Auxiliary verbs | 2.85 (7.79) | 3.74 (8.27) | 2.31 (4.99) | 1.05 (1.00-1.09) | .02 |
|  |  | Postpositions | 0.53 (2.04) | 1.19 (2.50) | 2.20 (3.21) | 1.17 (1.03-1.32) | .01 |
|  |  | Discrepancy | 3.26 (8.52) | 4.67 (10.31) | 1.76 (4.80) | 1.04 (1.01-1.08) | .02 |
|  |  | Tentative | 3.03 (7.87) | 4.64 (8.89) | 1.76 (4.80) | 1.05 (1.01-1.09) | .01 |
| H6 | Insomnia early | Auxiliary verbs | 3.38 (4.60) | 4.37 (4.38) | 7.24 (6.97) | 1.11 (1.03-1.18) | .004 |
|  |  | Discrepancy | 3.23 (4.65) | 4.45 (4.44) | 6.94 (7.21) | 1.08 (1.01-1.16) | .02 |
|  |  | Body | 0.39 (1.41) | 1.69 (3.57) | 0.38 (0.93) | 1.18 (1.05-1.33) | .004 |
|  |  | Home | 0.37 (1.37) | 1.32 (3.44) | 0.65 (1.84) | 1.18 (1.04-1.32) | .007 |
| H7 | Insomnia middle | Function words | 43.85 (22.97) | 49.62 (15.35) | 54.42 (17.24) | 1.02 (1.00-1.04) | .04 |
|  |  | Multifunction words | 3.03 (3.96) | 2.83 (3.78) | 1.89 (2.23) | 0.91 (0.82-0.99) | .04 |
|  |  | Negative emotions | 0.27 (1.53) | 0.07 (0.30) | 1.39 (2.62) | 1.22 (0.98-1.47) | .03 |
|  |  | Discrepancy | 5.77 (7.33) | 6.98 (6.43) | 8.49 (13.05) | 1.04 (1.00-1.09) | .04 |
| H8 | Insomnia late | Auxiliary verbs | 2.75 (4.85) | 3.35 (5.95) | 7.79 (10.42) | 1.06 (1.01-1.12) | .02 |
|  |  | Tense markers | 1.48 (3.18) | 0.76 (2.19) | 0.25 (0.91) | 0.83 (0.69-0.96) | .02 |
|  |  | Future tense markers | 0.28 (1.46) | 0.09 (0.39) | 0.00 (0.00) | 0.50 (0.23-0.84) | .03 |
|  |  | Humans | 0.20 (0.82) | 0.71 (2.41) | 0.00 (0.00) | 1.23 (1.00-1.47) | .03 |
| H9 | Somatic symptoms general | Sadness | 0.06 (0.54) | 0.36 (0.98) | 1.01 (2.67) | 1.54 (1.12-2.17) | .010 |
|  |  | Cognitive processes | 16.17 (11.32) | 19.29 (9.27) | 17.27 (10.93) | 1.03 (1.00-1.07) | .04 |
|  |  | Leisure | 0.21 (0.87) | 0.28 (0.92) | 0.52 (1.89) | 1.44 (1.04-1.93) | .02 |
| H10 | Feelings of guilt | Function words | 24.83 (23.59) | 41.26 (16.58) | 43.55 (11.07) | 1.02 (1.01-1.04) | .008 |
|  |  | Adverbs | 6.83 (9.90) | 11.50 (8.56) | 14.39 (12.54) | 1.04 (1.00-1.07) | .02 |
|  |  | Prepositions | 2.42 (4.41) | 5.33 (5.75) | 5.24 (4.17) | 1.06 (1.00-1.13) | .04 |
|  |  | Cognitive processes | 10.57 (14.52) | 15.30 (9.56) | 21.27 (13.81) | 1.03 (1.00-1.05) | .02 |
|  |  | Inclusive | 4.24 (8.55) | 6.34 (6.73) | 6.50 (6.52) | 1.04 (1.00-1.08) | .04 |
|  |  | Leisure | 0.05 (0.35) | 0.13 (0.52) | 0.47 (1.20) | 1.84 (1.04-3.09) | .02 |
| H11 | Suicide | Function words | 19.48 (22.32) | 42.48 (15.75) | 44.25 (17.64) | 1.04 (1.02-1.06) | <.001 |
|  |  | Verbs | 3.30 (10.62) | 15.08 (10.11) | 21.90 (8.03) | 1.08 (1.05-1.11) | <.001 |
|  |  | Auxiliary verbs | 0.39 (1.68) | 4.14 (7.46) | 5.27 (4.12) | 1.17 (1.08-1.28) | <.001 |
|  |  | Prepositions | 0.92 (2.85) | 7.07 (7.52) | 12.22 (10.04) | 1.19 (1.13-1.26) | <.001 |
|  |  | Multifunction words | 1.98 (9.79) | 8.75 (9.70) | 11.11 (8.70) | 1.05 (1.03-1.09) | <.001 |
|  |  | Tense markers | 0.30 (1.87) | 1.52 (2.88) | 3.09 (4.27) | 1.15 (1.03-1.29) | .01 |
|  |  | Past tense markers | 0.15 (1.25) | 1.09 (2.45) | 2.43 (3.99) | 1.24 (1.09-1.43) | .002 |
|  |  | Discrepancy | 0.94 (4.62) | 4.33 (7.42) | 5.74 (4.38) | 1.08 (1.03-1.14) | <.001 |
|  |  | Tentative | 1.16 (3.96) | 5.41 (7.76) | 2.96 (2.82) | 1.05 (1.00-1.11) | .04 |
|  |  | Relativity | 2.23 (6.13) | 9.21 (9.77) | 7.23 (7.26) | 1.05 (1.02-1.09) | .004 |
|  |  | Time | 1.12 (3.87) | 6.22 (9.16) | 4.03 (5.72) | 1.06 (1.01-1.11) | .01 |
| H12 | Anxiety psychic | Auxiliary verbs | 2.38 (5.09) | 7.93 (14.62) | 14.28 (21.90) | 1.04 (1.01-1.07) | .009 |
|  |  | Cognitive processes | 15.53 (16.28) | 21.90 (20.02) | 29.05 (21.57) | 1.02 (1.00-1.04) | .01 |
|  |  | Discrepancy | 2.70 (5.56) | 8.42 (14.86) | 14.92 (21.93) | 1.04 (1.01-1.07) | .008 |
| H13 | Anxiety somatic | Anxiety | 0.20 (1.08) | 0.12 (0.45) | 0.00 (0.00) | 0.57 (0.29-0.88) | .04 |
|  |  | Perceptual processes | 0.85 (2.53) | 0.95 (2.03) | 0.00 (0.00) | 0.78 (0.63-0.94) | .01 |
|  |  | Biological processes | 5.07 (6.71) | 5.63 (5.22) | 6.60 (6.60) | 0.93 (0.88-0.99) | .03 |
| H14 | Hypochrondriasis | Pronouns | 3.44 (7.83) | 4.55 (5.61) | 5.42 (6.57) | 0.95 (0.89-1.00) | .04 |
|  |  | Impersonal pronouns | 0.34 (1.36) | 0.30 (0.94) | 0.00 (0.00) | 0.66 (0.43-0.89) | .02 |
|  |  | Prepositions | 3.68 (7.30) | 5.41 (8.68) | 14.06 (14.03) | 1.05 (1.01-1.09) | .01 |
|  |  | Multifunction words | 3.26 (6.36) | 5.75 (8.60) | 13.39 (14.21) | 1.05 (1.01-1.10) | .008 |

Adjusted for age, gender, and depression severity.
