# Supplementary material for: Detection of Suicidal Ideation in Clinical Interviews for Depression Using Natural Language Processing and Machine Learning: Cross-Sectional Study
Source: JMIR Med Inform. 2023 Dec 1;11:e50221. doi: 10.2196/50221 (PMC10718481; doi:10.2196/50221)
Supplement: Multimedia Appendix 3 [file medinform-v11-e50221-s003.docx]

**Table A3.** The performance of detecting suicide risk based on verbal responses to the HAMD questions using random forest with leave-one-out cross-validation.

| Suicide risk | Participants | Total number  of participants | Targeted  participants | HAMD item | AUC (95% CI) | p | Sensitivity (%) | Specificity (%) |
| --- | --- | --- | --- | --- | --- | --- | --- | --- |
| High | All participants | 305 | 13 | 4 | 0.64 (0.53-0.74) | .01 | 53.85 | 59.93 |
|  |  | 305 | 13 | 8 | 0.66 (0.53-0.80) | .02 | 61.54 | 62.67 |
|  |  | 305 | 13 | 10 | 0.63 (0.51-0.76) | .03 | 61.54 | 56.85 |
|  |  | 305 | 13 | 11 | 0.89 (0.83-0.95) | <.001 | 84.62 | 83.56 |
|  |  | 305 | 13 | 14 | 0.76 (0.61-0.91) | <.001 | 69.23 | 69.86 |
|  | Participants with lifetime MDD | 197 | 13 | 4 | 0.60 (0.51-0.70) | .04 | 61.54 | 58.70 |
|  |  | 197 | 13 | 8 | 0.65 (0.51-0.79) | .03 | 61.54 | 63.59 |
|  |  | 197 | 13 | 11 | 0.85 (0.77-0.92) | <.001 | 76.92 | 77.17 |
|  |  | 197 | 13 | 14 | 0.76 (0.61-0.92) | <.001 | 76.92 | 77.17 |
|  | Participants with lifetime MDD  and unremitted depression | 109 | 13 | 6 | 0.67 (0.52-0.82) | .03 | 69.23 | 69.79 |
|  |  | 109 | 13 | 11 | 0.76 (0.63-0.88) | <.001 | 69.23 | 72.92 |
|  |  | 109 | 13 | 14 | 0.75 (0.59-0.91) | .003 | 69.23 | 68.75 |
| High & low | All participants | 305 | 69 | 1 | 0.68 (0.62-0.74) | <.001 | 60.87 | 61.86 |
|  |  | 305 | 69 | 2 | 0.65 (0.59-0.72) | <.001 | 59.42 | 59.32 |
|  |  | 305 | 69 | 3 | 0.60 (0.52-0.69) | .02 | 59.42 | 59.32 |
|  |  | 305 | 69 | 4 | 0.63 (0.55-0.71) | <.001 | 60.87 | 61.02 |
|  |  | 305 | 69 | 5 | 0.61 (0.54-0.68) | .002 | 62.32 | 62.71 |
|  |  | 305 | 69 | 6 | 0.59 (0.51-0.67) | .03 | 52.17 | 53.81 |
|  |  | 305 | 69 | 9 | 0.67 (0.60-0.74) | <.001 | 63.77 | 63.56 |
|  |  | 305 | 69 | 10 | 0.75 (0.69-0.81) | <.001 | 72.46 | 71.61 |
|  |  | 305 | 69 | 11 | 0.92 (0.88-0.95) | <.001 | 84.06 | 84.32 |
|  |  | 305 | 69 | 12 | 0.69 (0.62-0.75) | <.001 | 60.87 | 61.44 |
|  |  | 305 | 69 | 13 | 0.63 (0.56-0.70) | <.001 | 57.97 | 59.75 |
|  |  | 305 | 69 | 14 | 0.58 (0.50-0.65) | .04 | 55.07 | 55.08 |
|  | Participants with lifetime MDD | 197 | 64 | 1 | 0.60 (0.52-0.68) | .02 | 56.25 | 57.14 |
|  |  | 197 | 64 | 4 | 0.63 (0.55-0.72) | .002 | 62.50 | 61.65 |
|  |  | 197 | 64 | 5 | 0.63 (0.55-0.72) | .002 | 64.06 | 63.16 |
|  |  | 197 | 64 | 9 | 0.68 (0.60-0.76) | <.001 | 59.38 | 60.15 |
|  |  | 197 | 64 | 10 | 0.70 (0.63-0.78) | <.001 | 64.06 | 66.17 |
|  |  | 197 | 64 | 11 | 0.91 (0.87-0.95) | <.001 | 82.81 | 83.46 |
|  |  | 197 | 64 | 12 | 0.65 (0.57-0.73) | <.001 | 59.38 | 59.40 |
|  | Participants with lifetime MDD  and unremitted depression | 109 | 60 | 11 | 0.83 (0.75-0.91) | <.001 | 76.67 | 77.55 |
|  | Participants with lifetime MDD  and remitted depression | 88 | 4 | 10 | 0.68 (0.53-0.84) | .02 | 75.00 | 61.90 |
|  |  | 88 | 4 | 11 | 0.91 (0.82-0.99) | <.001 | 75.00 | 79.76 |
|  | Control participants | 108 | 5 | 11 | 0.70 (0.51-0.90) | .04 | 80.00 | 70.87 |

Participants with unremitted depression (HAMD-17 score ≥ 8).
